# The content and meaning of war experiences: A qualitative study of trauma and resilience among Liberian young refugees in Ghana

**DOI:** 10.1177/1363461520901636

**Published:** 2020-02-23

**Authors:** Felix Nyarko, Raija-Leena Punamäki

**Affiliations:** 1 University of Tampere; 2 University of Tampere

**Keywords:** Liberia, meaning of traumatic experiences, qualitative study, refugees, war trauma, young adults

## Abstract

Abundant research has shown that traumatic war experiences can interfere with the mental health and wellbeing of children and adolescents, but less is known about the subjective experiences and views of war survivors. The present study identified and described the different types of war experiences of young refugees in an African context and analyzed how they perceived the meanings and impact of war on their lives. The participants were 13 Liberian 25–35-year-old male and female refugees living in Ghana who agreed to take part in semi-structured interviews based on the life history approach. The transcripts were analyzed using a phenomenological method to detect themes incorporating multiple subthemes. The results revealed five main themes about war experiences, all negative in nature: pain and humiliation, loss of close relationships, horrific scenes, threats to life, and fleeing for one’s life. Concerning the perceived meanings and impact of war, the results identified six main themes. Three of them were positive in nature: increased awareness of life, compassion for life, and identification with those suffering. The negative main themes incorporated vivid horrific memories, deprivation of age-appropriate opportunities, and self-harm and destructive behavior. Our findings suggest that young war survivors may be highly motivated to participate in nation- and peace-building and should be provided opportunities to contribute to broader political and civic life.

## Introduction

War experiences can have broad impacts on child and adolescent development, but available research has mainly addressed the mental health consequences, such as increased symptoms of post-traumatic stress disorder (PTSD) and depression (Attanayake et al., 2009; Slone & Mann, 2016). In early childhood, core developmental tasks are related to gaining a sense of security and trust in other people, and in adolescence, it is important for individuals to create their own social relations and build their own sense of self and identity (Blakemore & Mills, 2014; Dahl, 2014). Traumatic war experiences may interfere with the accomplishment of these tasks, and therefore it is important to also study the developmental and existential influences of childhood war trauma. Accordingly, this qualitative study analyzed the experiences, consequences, and meanings of war among young adults, who had experienced the Liberian civil wars as children and adolescents and later settled as refugees in Ghana.

### War experiences and meanings

Children and adolescents living in war zones experience destruction, losses, atrocities, and threats to life, which can have devastating impacts on their wellbeing (UN Children's Fund (UNICEF), 2016). Ample evidence demonstrates that those in war zones experience high levels of mental health problems, such as depressive, anxiety, and dissociative symptoms and PTSD (Betancourt et al., 2010; Dimitry, 2012). Depressive and anxiety disorders have been found in about a third of children and adolescents living in war zones (Attanayake et al., 2009). PTSD, manifested in intrusive, avoidant, and hyperarousal symptoms and dysfunctional cognitive and emotional processes, seems highly common in African war contexts. For instance, more than three quarters of adolescents (78%) of a sample of war survivors in Uganda (*n* = 81) reported post-traumatic stress reactions of clinical significance (Schultz, Sorensen, & Waaktaar, 2012). In Rwanda, the National Trauma Survey (NTS) of 1547 respondents reported that overall 54–62% of children and adolescents (ages 8 = 19) met criteria for probable PTSD one year after the genocide (Neugebauer et al., 2009). In Middle Eastern studies among children and adolescents, a Palestinian study (*n* = 480) found that 53.5% scored clinically significant PTSD after a major war (Qouta, Palosaari, Diab, & Punamäki, 2012) and another Palestinian study (*n* = 197) found the clinical rate to be 70.1% (Thabet, Tawahina, El Sarraj, & Vostanis, 2008). In a meta-analysis of studies worldwide, the average rate of PTSD for war-exposed adolescents was 47% (Attanayake et al., 2009).

Research on mental health problems among war-affected adolescents has been criticized, and scholars have called for work to understand the personal meanings given to losses, destruction, and threats to life (Barber, 2013; McKay & Wessels, 2004). Qualitative studies have provided deeper understandings of the resources and survival skills of African former child soldiers (Boothby & Thomson, 2013; Harnisch & Montgomery, 2017). A follow-up study based on life-story interviews found that forcefully recruited Ugandan youth have a repertoire of meaningful coping strategies, a great variety of war experiences, and multiple sources of resilience, recovery tactics, and life philosophies (Harnisch & Montgomery, 2017). Almost all participants (94% of 36) do not mention war in their life stories and, when prompted, did not want to discuss their memories (Harnisch & Montgomery, 2017). Multiple avoidant coping modes included attempting to forget traumatic events and suppressing, numbing, and denying painful emotions, all hypothesized to serve the goal of continuing life without their past vulnerability (Harnisch & Montgomery, 2017). These findings are important because avoidance and emotional numbing are considered to be dysfunctional coping strategies in the western psychological literature (Pfefferbaum, Noffsinger, Wind, & Allen, 2014), whereas in war contexts, they have been found to serve as psychosocial adjustment. For example, a study found that Ugandan war-affected youth regard silence about their traumatic past as representing individual and community strength and cultural capital (Akello, Reis, & Richters, 2010). Qualitative studies have revealed highly nuanced, multiple, adjustment-focused, personal meanings of trauma and described the unique ways survivors continue their lives despite severe trauma (Denov, 2010; Efevbera & Betancourt, 2016). Research has also reported local idioms and meanings for mental health symptoms; for instance, Ugandan adolescents develop specific gestures and interactions in *kumu*—“sitting while holding one’s cheek with hands” or “not greeting people” (Betancourt, Speelman, Onyango, & Bolton, 2009, p. 251).

The results of these qualitative studies concur with growing evidence on resilience among war-affected children and adolescents (Tol, Song, & Jordans, 2013). Resilient survivors typically show good mental health and psychosocial functioning despite exposure to severe traumatic experiences (Masten & Tellegen, 2012; Werner, 2004; Punamäki, El Sarraj, & Qouta, 2001). Resilience reflects a capacity in recovering to normal functioning and even thriving after severe trauma. As a social and cultural phenomenon, resilience indicates that survivors are provided with access to multiple resources utilizing their strengths in harsh conditions (Ungar, 2013).

Qualitative studies have analyzed the cultural and social preconditions for resilience, documenting local protective factors in war contexts (Amone-P’Olak, 2007; Eggerman & Panter-Brick, 2010). These preconditions include social support, ideological commitment, finding new resources in oneself and community, and successful efforts to make sense of and find meaning in trauma and suffering (De Nutte, Okello, & Derluyn, 2017; Punamäki, 2014; Klassen et al., 2010; Punamäki, 2000). For instance, a participatory study identified 40 meanings of resilience among war-affected Ugandan children, primarily possibilities to achieve favorable goals and spiritual and material success in life, increase self-reliance, hold high morals, and enjoy good health (Vindevogel et al., 2015). Respect for human rights and protection of vulnerable groups in war conditions are examples of resilience-enhancing social frames (Punamäki, 2014; Punamäki, Qouta, Miller, & El Sarraj, 2011).

### Study aims

This study was aimed at understanding war experiences and subjective perceptions of their impacts and meanings in an African context. This study analyzed the experiences of Liberian young adults who, along with their families, went through atrocities during the civil wars and sought refuge in a neighboring country, Ghana. The first research task was to describe and identify the different types of the participants’ war experiences. The second task was to analyze how these young war survivors perceived the impacts of war on their current lives, constructed and made sense of these impacts, and gave meanings to their war experiences.

## Methods

### Participants and procedure

The study participants were 13 Liberian young-adult refugees living in Ghana: six women and seven men, all 25–35 years old. Four had completed high school, three had attained bachelor’s degrees, and one had no formal education. Five were high-school dropouts.

Participants were interviewed in a refugee camp in the Central Region of Ghana. Convenience and snowball sampling procedures were applied by approaching the camp manager and headmistress of a vocational school to gain access to potential participants and then asking about the interviewees’ networks. Two considerations determined when the sample size was sufficient: generation of in-depth, varied narratives of war experiences and associated meanings based on the saturation principle (Moustakas, 1994), and recruitment of equal numbers of men and women.

The Ghana Refugee Board, Ministry of Interior, provided approval of the study methodology and methods (RB:0140/vol2/18). The researcher (the first author) informed the potential participants about the study aims, confidentiality, and ethical rules and then conducted the interviews. The participants gave oral consent recorded as a part of the interview procedure. The interviews were conducted in the school setting in rooms that provided privacy.

### Interviews

The semi-structured interview method was selected because it allows participants to tell their stories, memories, feelings, behaviors, and subjective experiences (Moustakas, 1994; Silverstein, Auerbach, & Levant, 2006). The interviews were conducted in English, Liberia’s official language, and began with a short background chat, to make the participants feel comfortable sharing their experiences, emotions, and thoughts. The participants spoke of the war experiences and their meanings in modified, semi-structured, life-history interviews with open-ended questions focused on childhood and family memories, important life events, and experiences during the civil wars in Liberia, and life as a refugee. The discussion on the war started with very general questions (e.g., “What was it like to live as a child or an adolescent in war?” “Have these experiences influenced your life?”). The young adults’ answers were followed by prompting inquiries (e.g., “Can you please give an example?,” “How and why there were impacts of war?,” “What are your best and worst memories related to what you told?”).

### Data analysis

The interviews were transcribed and subjected to detailed qualitative analysis. Main themes composed of multiple subthemes were identified in four stages following the principles of phenomenological content analysis (Hycner, 1999; Moustakas, 1994). First, the first author repeatedly read the 13 transcribed interviews to gain familiarity with their general expressions, messages, and contents and then selected all the text extracts referring to war experiences and the meanings given to them. The unit of analysis to identify relevant material was a whole interview, not only the responses to specific questions about war experiences and consequences. For instance, discussions on childhood and family often evoked memories of the atrocities of the Liberian civil wars, and conversations about current experiences in the refugee camp evoked memories, emotions, and thoughts about the war. Second, the two authors independently categorized these text extracts from the interviews and labelled the identified subthemes according to their distinct contents and messages. The subthemes closely corresponded to the text, reflecting the unique narratives, memories, experiences, and meanings. Third, the two coders compared the contents and logics of their independently identified subthemes and negotiated to agree on specific, additional subthemes. Fourth, the first author grouped the identified, agreed-upon subthemes into the main themes, and the two coders again conferred about the number, names, and contents of the final main themes. Very similar subthemes were combined to reveal the structure and content of the themes. To maintain the validity of the reported material and transmit the voices of participants, direct quotes from the participants were reported in the results.

## Results

### Themes of adolescents’ war experiences

The analysis identified five main themes of war experiences: reflecting on pain and humiliation, loss of close relationships, horrific scenes, threats to life, and fleeing for one’s life. Table 1 presents the subthemes constituting these main themes. The results documented a variety of experiences including unique meanings, vivid memories of cruelty, and cultural, social, political, and psychological symbols. Common to the recollections, stories, and narratives were highly emotional tones of sadness, rage, fear, despair, and longing. In the following, we present the main themes and their subthemes.

**Table 1.** Themes and subthemes of the experience of war.

Themes	Subthemes
Pain and humiliation	Pain due to suffering and humiliation of loved ones
	Pain of being betrayed
	Humiliation and loss of human dignity
	Physical pain and lack of remedies
	Vivid images of humiliation
Loss of close relationships	Impossibility for parents to protect their children
	Loss of loved ones
	Loss of guidance and direction for the future
	Loss of home
	Strong sense of neglect and lost security and abundance
Horrific scenes	Sexual assault and torture
	Witnessing cruelty and injustice
	Human-caused pain
	War horrors beyond words
Threats to life	Fear of abduction and conscription as child soldiers
	Forced to act against one’s own morals
	Forced to act against one’s own people
	Helplessness due to inability to act
Fleeing for one’s life	Being haunted everywhere
	Endless walking to escape death
	Near misses of death and surprise at survival
	Starvation and fear of death

#### Pain and humiliation

The participants recounted inhuman, shameful, and humiliating experiences during the wars when fleeing from Liberia and becoming refugees. They told how their enemies systematically used dehumanization, torture, and other cruelties to instill fear and intimidate people. The analysis identified five subthemes: psychological pain, shattering of human dignity, betrayal, humiliation and physical pain carrying the fear of death, and intrusive, vivid images of pain and humiliation.

Psychological pain resulted from witnessing the humiliation and suffering of loved ones and being betrayed by other humans. Feelings of betrayals burdened the participants, who related that their trusted relatives and neighbors turned out to be enemies and traitors, killing and abusing people with whom they had lived together for years. The participants’ sense of reality was undermined because they could not believe that evil of this magnitude was possible:

My father was a police colonel. He was betrayed and stabbed in the back by his colleague, a man who we use to called uncle when he came into our house. The man gave up my father to be killed in order to protect his family. My father was taken in a pickup truck to a firing squad and was killed. We escaped and went to my mother’s hometown and stayed there for some time.

Physical pain typically involved fears of being wounded, tortured to death, and neglected without treatment for unbearable pain. The participants recounted vivid memories of atrocities and pain inflicted on themselves and their families, which still caused them pain and insulted their dignity:

My father asked us to run because there was a plot to kill him. Before we could escape, they caught us and tied our legs and hands. We were lying on our bellies. They were just kicking and stepping on us. They were also slapping our faces and hitting our mouths while others were whipping us with sticks on all parts of our bodies. The officer who executed my father was asked to also execute us, but as he was taking us away to do so, he decided to set us free.

#### Loss of close relationships

The multitude of losses formed a core experience of war survivors and refugees. The five subthemes of loss described the loss of security, protection, and guidance in life, indicating a deep sense of being left alone and neglected in a huge world. Many stories reflected the painful realization that parents could not protect their children from atrocities. These losses and the accompanying unsafety still dominated the participants’ lives.

Several participants recalled how they lost track of or were forcibly separated from family members while fleeing terror and violence. Many were still uncertain about the whereabouts or the fate of close family members. The uncertainty of whether loved ones were alive or dead deepened the participants’ grief and pain:

My father, realizing the cruel situation at hand, painfully advised my mother to escape with us to a place of safety, which was La Cote d’Ivoire. Unfortunately, the car we were going to escape in was taken away by the soldiers. I lost track of my mother and siblings running to the place of safety. My father was killed by the soldiers behind us. As of now, I do not know the whereabouts of my mother and siblings, whether they are dead or alive.

The narratives reflected the profound sense of loss of family as a guiding, loving, protective shield against a dangerous world. The participants described how they felt lonely and vulnerable in the unsafe world with no one to guide and support them through life:

Somehow, it has stopped me from enjoying life because had it not been the war which destroyed my family, burned down our home and everything they had worked for, I would have gone to school, and now my parents would be alive to take care of me. It is just like there is an umbrella, and everybody is under that umbrella, but all of a sudden, the umbrella was taken away, so you have to endure the scorching sun*.*

The destruction of home resulted in both symbolic and material losses. The participants described home as their most intimate place where they cherished rich memories. Many saw their homes burned and destroyed and vividly felt that their nurturing family relationships, identity, and sense of security also were burned and still felt the pain of loss in the flames:

My aunty was prepared to take me through university, but the rebels took away her money and everything she had worked for, so she was not able to fulfil her promise. This has been a major setback for me. I was hoping to become a doctor, but now all hope is lost. It took away my dream, my family, our house, my aunt’s business, and now I am here at the camp struggling to undergo vocational training. My dream of becoming a medical doctor was shattered.

#### Horrific scenes

The participants’ narratives revealed constant, intrusive, involuntary memories of dreadfully violent acts of torture and atrocities. They included flashbacks of rape and other sexual assaults, seeing dead bodies and body parts, and hearing the screams of dying and severely wounded people. Witnessing horrors and torture of family members evoked especially powerful feelings of helplessness, rage, shame, and fear. Many horrific scenes, such as people being beaten with sticks and killed in cruel ways, remained imprinted in memory:

My family and I were thinking whether to stay or run when the war broke out. The rebels entered our house and started beating us. My brother died from the severe beatings they subjected him to. I was abducted and locked up with a few girls in a place, and these rebels would come any time they pleased to beat and rape us in turn. They denied us food and freedom while our parents were separated from us. We were at their mercy as they were taking the law onto their own hands. We managed to escape by sheer luck.

The narratives of human-caused pain often involved deep despair and shattered worldviews. The participants related their devastating realization that human life was the cheapest commodity because killing a fellow human meant nothing to the armed groups. It was shocking to learn that their fellow humans, even their enemies, were impervious to the normal emotions of empathy, compassion, and reason. The indiscriminate killings and torturing of civilians became routine in the Liberian civil wars:

My aunty and I were captured when the rebels entered the house. My aunty was beaten and sexually molested in turn with a gun pointed at her and me. After that, she was shot multiple times in her belly and died in a horrifying manner. This was right in front me, and I have not seen a person go through such pain before dying. I was tortured, but luckily, I was the only person who survived in my family. I am now alone in this world, and now when a person close dies, the memories keep coming back.

#### Threats to life

The participants described living in prolonged fear and terror, which threatened their physical and psychological wellbeing. Abduction and conscription as child soldiers were one of the most troubling fears, involving the impossible dilemma of whether to kill a fellow human or even a family member or be killed oneself. Some participants told how rebels abducted, tortured, threatened murder, and intentionally drugged them to force them to join their gangs. Although many were aware that they had been forced to act against their own people and their own moral principles, they still expressed deep regret, despair, and shame:

I was also forced to harm others as a child soldier. If you say no, then you are an enemy of the revolution, and you will be conscripted to join the group to fight. We stayed in the bush and did not come to town because if you did, you would be forced to fight. I was induced by drugs to become numb to cry the battle cry. This will make you insensitive to taking up arms and fighting. You will see an elderly woman, and you are asked to strip her naked, kill this man, and do other horrible stuff. This is an abuse in its own right.

#### Fleeing for life

Many war experiences evoked the feeling of being constantly hunted wherever the survivors tried to hide. Many participants told that they had to walk for days, throughout the night, and in the heat of the day at the mercy of the weather. They ran for their lives to get to a place of safety as the violence and warfare drew closer. They constantly feared running into the lair of an armed group or being trapped between warring factions:

The journey through the bushes and forest was dangerous, with people vomiting and trembling in pain. I walked for days and night without food but drinking unhygienic water and eating wild fruit before I got to the border of Cote d’Ivoire. I remember walking into a rebel-held area in the bush. They took me to a camp in the bush where I met young girls who had also been captured. We stayed for days without food, and any time they had an urge for sex, they came for us and sexually abused us in turns until the camp was invaded by peacekeepers, and we escaped.

A prominent experience was starvation. The participants told how they had to live on wild fruit to sustain themselves or boil leaves to eat to gain the strength to keep going. They witnessed how lack of food and water led family members to death:

This is how I picture the war, how we were walking in the bushes for days and months with the hope to find a place of safety. We had to uproot cassava and eat them raw just to survive. How people were looking for food and water, which was ever not there, my brother—it brings tears to my eyes; it was not easy at all. Many of the people with whom were walking died because of starvation and dehydration.

The participants told that while fleeing, they often narrowly escaped death. They described a feeling of unreality and surprise at being alive amid atrocities and death. They did not know where they were going and were filled with fear while walking endlessly in the bush and forest. Their narratives told of their single-minded determination and instinctive desire to survive:

My grandfather had a cattle farm at that time, so when the war broke out, we decided to go there because we could not find our mum. We thought she would be there. When we went there, the rebels were on the farm, and they had killed my grandfather and were killing the cattle. In the process of running through the forest and bushes, a stray bullet missed my hand but scratched my left arm. I got wounded while running through the bush and forest away from the rebels. I stepped on a twig, and it went into my left leg. It was a deep wound, which lasted for six months because there was no medication.

### Themes of the impacts of war experiences

The results presented six main themes about the impacts and meanings of war in the Liberian young adults’ lives. The participants pondered their life histories from different perspectives and found depriving, harmful, and burdening impacts and enlightening, strengthening, and instructive meanings. As seen in Table 2, three main themes concerned negative impacts and suffering: vivid horrific images, deprivation of age-appropriate opportunities, and self-harm and destructive behavior.

**Table 2.** Themes and subthemes of the perceived meanings and impact of war on one’s life.

Themes	Subthemes
Vivid horrific memories	Unending, negative visuals of cruelties
	Carrying horrible scenes within oneself
	Everyday events triggering horrors
	Avoiding painful reminders
Deprived of age-appropriate opportunities	Lost hopes, dreams, and desired life
	Vivid image of lost potential
	Deprivation of life fulfillment and enjoyment
	Only grim alternatives available
	Wrong choices due to economic hardships
Self-harm and destructive behavior	Dysfunctional attempts to regain control
	Reckless and dangerous behavior
	Counterproductive coping modes
Increased awareness of life	Difference between the past and the present
	Struggle against bitterness
	Awareness of new developmental gains
	Awareness of injustice
Compassion for life	Gratitude for being alive
	Appreciation of social bonds
	Religion as a source of strength
	Strength from harsh experiences
	Urge to understand
Identification with those who suffer	Deep understanding of what starvation means
	Empathy and need to help those suffering
	Appreciation of help and support
	Responsibility for humankind

#### Vivid horrific memories

The colossal impacts of war experiences seemed to be the involuntary, constant, and never-ending presence of atrocities and persecution in the young adults’ minds and memories. The participants were children during the Liberian civil wars, and their life trajectories were filled with painful losses and trepidation. Acts of violence, brutality, humiliation, losses, and atrocities still haunted them. Almost all the participants reported recurrent, intrusive, distressing recollections in the form of flashbacks, nightmares, and unpleasant bodily sensations. Many stated that carrying these horrible scenes of war within them made them weak, fearful persons. These disturbing and disorienting memories were still very scary and often unbearable:

I used to have awful, frequent dreams about my aunt who was brutally killed during the war. It affected me because I was with her at that time, and it was a traumatic experience for me. Until today, it still horrifies me. If I recall it, I cannot sleep at night.

I was nearly raped by one of the rebels in front of my mother, but—I don’t know what happened—all of a sudden, one of them, he asked us to run out of the place. I remember my mother went out one day to look for food, then she met some of the rebels, and they asked for money, but she did not have any, and they beat her to death. My brother was shot when some rebels came to ask for money and food. That scene still horrifies me today. My aunty died because she attacked the man who killed her son and was shot. It looked like a horror movie any time I recall it. If I remember it, it brings pain and tears.

The participants further described how everyday encounters in the refugee camp could reactivate their painful memories, interfering with their social relationships and schoolwork. The triggered, unsettling memories often led them to avoid certain situations, signs, and events that could evoke memories of the past. The participants commented: “I stay away from crowded areas.” “I don’t like seeing blood or people fighting.” “I avoid arguments or all risks of getting angry.” A few participants even told about daily encounters with their former victimizers in the refugee camp in Ghana, which made them fearful and triggered intrusive memories.

For some participants, their past, shameful memories and the “carrying [of] horrible scenes within” them signified moral dilemmas and a kind of personal trial. Many of those participants had been abducted and forced to become child soldiers and were trying to recover from the atrocities they had committed and witnessed. One participant stated: “I want to forget my life as a child soldier and get accustomed to my new life, but I am struggling. I did not have a choice back then, and I did what I did as a child.” The participants expressed remorse and told about the overwhelming burden of guilt and sorrow they felt for committing such horrific atrocities. “I feel ashamed of myself and of what I did. I do not imagine my life if I could claim my lost humanity back,” one participant stated.

#### Deprivation of age-appropriate opportunities

This theme included the multiple reasons the participants felt regret, sorrow, and anger about their current lives. They strongly believed that the Liberian civil wars and being refugees had destroyed their opportunities for personal and professional development. Often, war ruined the participants’ hopes for education, although they tried to continue to study in refugee camps. However, constant fears and intrusive war memories interfered with successful learning, and some stated that the tragic loss of their parents and material possessions restricted their schooling, making school fees unaffordable. The participants were highly aware of the loss and vividly imagined how their lives would have been different without their war experiences:

The war robbed me of my education and made poverty come to my family. If it had not been for the war which destroyed my family, I would have gone to school and attained a significant level of education with the support of my parents. But now, it is all a dream.

Yes, I should have completed my first degree and be working by now. Sometimes I have to sleep without food, and I continue to struggle to finish vocational training. It is painful to see my age mates who are Ghanaians driving their own cars, while at my age, I am still struggling with what to eat. It is not fair at all. If there had not been the war, by now, I would also be enjoying life and working as a professional nurse. If I enter into prostitution today, it will be because I need money to survive.

The deprivation of possibilities led some participants to state that life had offered them only bad and grim alternatives. Among the worst were engagement in prostitution and promiscuity for survival. The participants desperately attempted to understand the grim fates and choices of themselves and other refugees. They pondered, for instance, whether girls abused during the war later engaged in reckless practices of their own will to find validation for the past. Their present, challenging conditions made them vulnerable to falling prey to the ills of society:

I remember one time in the camp, my sisters were threatening my mum to enter into prostitution because they believed that those girls were not worse than they, and they needed money to take care of themselves. My mother broke down in tears and began to beg them not to make that decision. I felt terrible, and it was the worst moment of my life in the camp because it made me cry the whole day, and I felt like ending it all. I feel pain in my heart as I recall these things.

#### Self-harm and destructive behavior

A number of the participants analyzed the war impacts in terms of the traumatization and the profound changes it caused in their future prospects. Consequently, they disregarded the dangers and severe consequences of their reckless actions. They noted that numbing their feelings was necessary to survive the war but later made them ignore their own wellbeing and self-respect. Self-harm and destructive behavior reflected failed attempts to master pain and shame, resulting in dysfunctional coping strategies, such as alcohol and drug use. For former child soldiers, drug abuse was partly a habit acquired during the war:

The pain of loss of my family and the dreadful things witnessed horrified me when I came here. I lost hope and a sense of direction. The future did not mean anything to me because it was bleak to me. I joined bad company, and I engaged in bad activities like drugs and alcohol. I was even invited by a friend to join their armed-robbery gang because at that time, I did not have any meaning in life. I thank a pastor who guided and counseled me through such reckless behavior. I was indulging myself because of traumatic experiences.

I knew how to use a gun because I used to be a child soldier fighting for the rebels. Even during the war, drugs and some concoctions were mixed for us to drink to become numb to human feelings. All manner of atrocities against people were committed by my group and me. When I got here to the camp, I continued taking drugs because I needed it to forget about the horrible things I did. I finally joined a group, and we undertook a lot of armed-robbery attacks until our leader was killed in one of our operations. I was fortunate to escape, and that was the turning point in my life.

As shown in Table 2, two main themes of impacts of war revealed positive experiences: compassion for life and identification with those who suffer. The subtheme of increased awareness of life involved both negative and positive realizations of the impacts of war.

#### Increased awareness of life

The participants repeatedly stated that the wartime events and refugee experience had been their cruel teacher in life. These experiences forced them to become aware of harsh realities and uncertainties. Some participants told that they had lived in comfort in Liberia and now faced huge adjustment difficulties:

I was having it all, real wealth, and my family could afford anything for my siblings and me. We were living in a wonderful neighborhood. We had nannies, and my parents treated us as royals. My father would take us to school and back in his car. Now I found myself in a refugee camp, struggling to feed and serve my son and myself. It is difficult to come to terms with this situation because I was not prepared for what am going through. The war has changed my life and robbed me of my dream of becoming a nurse. I have lost everything, and I can forget it all.

The participants were aware of and struggled against the danger of becoming bitter, grudge-filled, and revengeful. They strove to break the chains of the hurtful past. They felt that liberating themselves from the unjust past helped make something meaningful of their lives. They were pleased to discover new aspects of themselves, build new relationships, and find new safety in life:

My experience in the war has strengthened me to do something for myself. I would have been wallowing in pain and engaged in self-destruction, throwing my hands into despair and blaming the perpetrators all my life. They did what they thought was right in their sight, but the Lord saw me through this gruesome experience. I am not going to let myself be drawn down by past events. No matter how traumatic it was, I will press on. I want to make use of every opportunity that I get to better my life because what is ahead of me is greater.

On the negative end of increased awareness are observations of deep injustice, abuse, and cruelty. War experiences have increased some survivors’ awareness of petty-mindedness and selfish motivations of other people and made them realize the huge gaps that separate them from their peers living in peaceful societies:

Yes, the war has shaped my life. I always think about the things that I went through, and it hurts like a fresh wound. It never heals. When I see young women of my age in Ghana going to work and taking care of themselves, I feel pity for myself. I even want to further my education, but there is no one to help me. The men just come around you when they need sex, so they do not respect you. It hurts when there is no support.

#### Compassion for life

This theme incorporated positive shifts to renewed appreciation for life, gratitude for surviving atrocities, and spiritual revitalization. The survivors’ hard experiences taught them to be considerate and sympathetic to other people and to value their new sense of purpose in life and compassionate feelings:

It was a tedious thing, and I am praying that nobody enters into such a situation. However, it has been helpful in a way because right now, it motivates me that I have still made it as far as I have in life. Sometimes you wouldn’t even know where the next bullet would be flying from, but by His grace, I survived. You would see most people dying, and everybody was running for survival. My parents and siblings were nowhere to be found, and I was left alone, but only God has the answer. I just had to follow others, so I could get something to eat in a day.

Religion functioned as source of strength for many participants, providing a framework for understanding what happened in war. Engaging in religious activity gave them inspiration, spiritual safety, and motivation to cope with the ills at the refugee camp and to help others who struggled with their pain:

My family and I survived the terror by an act of God. I remember one time, the place we were hiding, the rebels came around and started to shoot, and my mother told us all to lie down flat. It was a scary thing, and I did not expect us to survive, but by His grace, they left. Any time I remember that moment and the horrific things I saw in my escape route, I read the Bible and listen to gospel music.

I was having images of the killings and the evil things I saw back in Liberia. It was like I was watching a movie, so I decided to go to church. I started attending church and praying, and all the images and nightmare I was having stopped.

The participants recounted that surviving atrocities had strengthened them and increased their trust in their ability to endure. “If I am alive, nothing can bring me down,” a participant said. They felt surprise and gratitude and cherished the feelings of joy, mastery, and meaningfulness. One stated, “As human beings, you do not know how strong and resilient you are until you are forced to bring that hidden strength forward to make a change in your life”:

It has not changed anything but rather made me strong and wise. This means nothing can bring me down and hinder me in the pursuit of my future life. I could pass through bullets. I did not die. The bullet hit my hand. I did not die. That means God has a purpose for my life. That is why he made me survive through this turbulence. I always keep motivating myself and push myself to greater heights.

The participants expressed an urgent need to understand their own fate and the reasons for the excessive violence during the civil wars. They wanted to learn their perpetrators’ motivations for their inhumane acts and willingness to cause pain to fellow citizens. One participant stressed, “I want to have the opportunity to confront my torturers directly and ask them why they subjected my family and me to that humiliation and abuse*.*” Many participants also wanted the perpetrators to acknowledge and assume responsibility for their cruel deeds and apologize for their wrongdoing.

#### Identifying with those who suffer

A number of the participants stated that their own painful experiences gave them a deeper understanding of what suffering meant. Their own persecution and abuse helped them develop empathy for underprivileged people. Their experiences of starvation lead to deep empathy for others who suffered privation. Many narrated how, while fleeing and seeking safety, only those who got help from others avoided death. This memory reinforced their desire to help people who were suffering:

The experiences in the war have made me to want to help those in need. I know what it means for a person to tell me that they have not eaten for days. I remember that during those times, I walked for days, and there was no food around, so we uprooted cassava and ate them raw just to survive. Sometimes when I picture the war and how people were looking for food, it was not easy at all.

## Discussion

This study generated empirical knowledge of the contents and meanings of war experiences for Liberian young adults living as refugees in Ghana. The findings revealed five main themes characterizing their war recollections: pain and humiliation, loss of close family members, horrific scenes, threats to life, and fleeing for one’s life. All these experiences were highly negative, but when the participants evaluated the impacts and meanings of the war experiences on their lives, two of the six main themes were positive: deepened compassion for life and empathy for others who suffer.

The interview method successfully enabled listening to the Liberian young adults’ own voices and experiences. They presented diverse, touching, nuanced views of their vulnerabilities, strengths, failures, and successes as war victims and survivors. The findings are consistent with the dark views of childhood and adolescence in war conditions in Uganda (Schultz et al., 2012), Rwanda (Neugebauer et al., 2009), and Sierra Leone (Denov, 2010), reflecting the psychological, social, and physical suffering of traumatic stress. However, the findings also demonstrate the participants’ resilience and motivation to gain strength and break down violent, traumatic, vicious circles, supporting earlier studies on African war survivors (Efevbera & Betancourt, 2016; Harnisch & Montgomery, 2017).

### War experiences haunting the present

The magnitude of violence, atrocities, torture, and horrors was overwhelming in the war memories of the Liberian young adults. They described how scenes of abductions, killings, dead bodies, and wounded people constantly haunted them. The participants reported experiencing nightmares, flashbacks, dissociative states, and strange bodily feelings when facing reminders of the horrors. The participants’ narratives thus depicted the core experiences of involuntary, uncontrollable phenomenon typical of intrusive symptoms of PTSD. The contents of these descriptions appear to match the core experiences of war survivors suffering from PTSD found in African contexts (Geltman, Grant-Knight, & Mehta, 2005; Schultz et al., 2012). These findings are important because intrusive symptoms can interfere with young adults’ social and intimate relationships, learning, and general wellbeing (Elbert et al., 2009; Yoder, Tol, Reis, & de Jong, 2016). Peers and other significant others may be frightened by the sudden, absent state of mind, startle attacks, and fear expressions that characterize PTSD (Punamäki, 2002).

Losing family members and witnessing cruelties toward them during the war were very painful to the Liberians survivors. As young adults, they still very vividly described how vulnerable, alone, and helpless they felt as children when enemies killed their fathers, mothers, siblings, and friends. They felt that no one protected them, and they were rejected, lost, and forgotten into a huge, dangerous world. These results are consistent with evidence that parental loss and separation pose a high risk for depression and anxiety among Sierra Leonean children (Betancourt et al., 2008; Betancourt et al., 2010) and PTSD among Sudanese refugee children (Geltman et al., 2005).

The participants insightfully analyzed how separation from, or loss of, loved ones had decisively influenced their wellbeing, social opportunities, and personality development. They were children during these losses, so their dependency and emotional openness made them vulnerable, and the cruelties and stressors influenced their later development. These self-observations support findings that the traumatic loss of parents negatively influences children’s coping strategies, psychological defenses, and adjustment abilities (Badri, Crutzen, & Van den Borne, 2012).

The participants showed severe distress and strong emotions when they described the experiences of threats to their lives and fleeing for their lives. The memories of being forced to act against their own moral views and their own people seemed to be the most painful. The history of being abducted and conscripted as child soldiers stole their innocence. This finding helps understand why the risks of PTSD, depression, and anxiety are so high among conscripted child soldiers (Humphreys, 2009; McMullen, O’Callaghan, Richards, Eakin, & Rafferty, 2012). Overwhelming feelings of guilt, shame, and regret can prevent healing and recovery from a traumatic past (Betancourt et al., 2010; Denov, 2010). Yet, the experiences can also increase compassion and affiliation with fellow humans’ suffering, as the present study showed.

Sexual assault was a highly distressful and shameful experience among the interviewed young women. Research in Rwanda and Sierra Leone has confirmed that exposure to sexual violence causes severe suffering, such as PTSD and reproductive health problems (Betancourt et al., 2010; Hogwood, Mushashi, Jones, & Auerbach, 2017; Neugebauer et al., 2009). Human rights organizations have repeatedly condemned the use of rape as a brutal weapon in contemporary warfare. Armed groups systematically and extensively commit rape and sexual violence to dehumanize, humiliate, and dominate adolescent girls and make their communities vulnerable (African Rights, 2004). The assaulted women can suffer their whole lives, and the experience can undermine their interpersonal relationships, sense of security, and identity formation (Hogwood et al., 2017). Sexual assaults often destroy young women’s ability to marry due to their own lost desire and the stigma placed on them. One participant expressed: “I have vowed not to get married because as a woman, my pride has been soiled, and therefore, I have a strong hatred for men and sexual affection after being raped during the war.”

### Negative and positive impacts of war

Deprivation of age-appropriate opportunities, such as receiving an education, forming a family, belonging to a community, and dreaming about the future, constituted a core theme of the impacts of war. The participants told how sometimes simply encountering Ghanaian young couples made them sad because their easy togetherness reminded the participants that the war had destroyed their own hopes for careers and families. As refugees, they often felt like second-class citizens and were highly aware of their limited choices due to economic and legal barriers.

Research has shown high levels of risk, self-harm, and antisocial behavior among young African war survivors, especially former child soldiers (Sharma, Fine, Brennan, & Betancourt, 2017; Ullman, Relyea, Peter-Hagene, & Vasquez, 2013). Similarly, the participants in this study described high-risk behaviors as a consequence of the war, including prostitution, armed robbery, and alcohol and substance abuse. The participants liked to reflect on the reasons for their behaviors. Some understood their dangerous behavior as dysfunctional attempts to cope with, forget, and numb the horrifying memories of war. Others explained that they engaged in risky, criminal or self-harming behavior due to a lack of available alternatives.

In accordance with the theoretical premises of research on resilience (Masten & Tellegren, 2012; Ungar, 2013), the Liberian young adults demonstrated mental growth, increased awareness, altruism, and deepening compassion for others, and they emphasized the beauty of life. Their traumatic war experiences contributed to the crystallization and awareness of their most important values and a deep, comprehensive understanding of suffering. The horrendous war experiences motivated the participants to make extra efforts to help and empathize with their fellow humans facing similar suffering. Their choice of an empathetic, compassionate life philosophy was based on gaining a deeper meaning of life, a profound sense of the human condition, and personal understanding of experiences such as hunger, mortal danger, humiliation, and cruelty.

Similar transformations of social and human values have been documented after terror attacks in western countries (Poulin, Silver, Gil-Rivas, Holman, & Mclntosh, 2009). In the present study, some participants explained that feelings of empathy were also important to their own physical and psychological integration because compassion for others liberated them from the chains of the hurtful past and protected them from grudges. Traditional African value systems view humans as social creatures knit together in webs of collective solidarity (Stark, 2006). Consequently, despite or even because of their experiences of horrific human rights violations, the participants felt they had a moral obligation to extend humanity even to their perpetrators. In this way, human dignity and personhood can be enhanced to strengthen bonds of communal unity, or as expressed by Mbiti (1991, p. 171), “The individual can only say, I am because we are, and since we are, therefore I am.”

Seeking religious and spiritual meanings were very important to ameliorating suffering. Research has confirmed that religious commitment functions as an effective way of coping, enhancing psychological adjustment and buffering against risk behaviors, such as prostitution and alcoholism (Ano & Vasconcelles, 2005; Ashby-Wills, Yaeger, & Sandy, 2003; Farley, 2007). In Africa, the church has been active in promoting reconciliation and creating new forms of bonding, support, and solidarity among former enemies. Traditional, local spiritual rituals are employed to heal war trauma and animosities. For example, former Mozambique child soldiers have been reintegrated into their communities through traditional cleansing ceremonies, contributing to psychological recovery, transforming self-images, improving self-esteem, and encouraging acceptance by community members (Boothby, Crawford, & Halperin, 2006).

The study has several limitations. As a small-sample qualitative study, it is not possible to generalize the findings across other African countries exposed to civil wars, for example, Rwanda, Sierra-Leone, or Mozambique. Different political, cultural, and military contexts exercise unique impacts on civilians’ experiences, and this study reflected those of Liberian refugees settled in Ghana. We applied a qualitative and phenomenological paradigm that is based on the interviewer’s openness, setting aside presuppositions, biases or earlier theoretical concepts of the phenomenon. However, we structured the interviews according to a life history approach, asking about the participants’ childhood and adolescent experiences and implicitly expected to hear memories of war. This may have introduced biases into the narrative. The interviews were conducted in English, which is the official language of Liberia, but communication using the participants’ mother tongue would have been ideal. This was not feasible due to budgetary and logistical limitations, given that there are 27 indigenous languages spoken in Liberia.^1^

## Conclusion

This study of young Liberian refugees in Ghana found both highly negative and highly positive life experiences among Liberian young war survivors. These findings are important for professionals working with war-affected children and youth, politicians reconstructing post-conflict societies, and victims and survivors themselves. Trauma research has recognized the potential for resilience and strength, and our study documented survivors’ urge to seek meaning, understanding, and recovery. Our study may thus help present the life of young refugees beyond models portraying these populations as helpless, passive victims without capabilities or inspiration for their lives. However, the study also recognized the refugees’ lack of opportunities, limited or no access to education, and extreme deprivation in the refugee camp. These adolescents able to survive the torrent of the civil war represent the future generation of Liberia. Great attention, therefore, must be given to their future potential to allow them to contribute to their own lives and the future of their nation.

## Note

1 See www.ethnologue.com/country/LR
